# Hepatoprotective evaluation of benzoylated emodin derivatives: Integrating bioinformatics and *in vitro* studies

**DOI:** 10.37796/2211-8039.1705

**Published:** 2026-06-01

**Authors:** Firdayani, Putri Hawa Syaifie, Siska Andrina Kusumastuti, Muthia Rahayu Iresha, Wirawan Adikusuma, Dhecella Winy Cintya Ningrum, Etik Mardliyati, Ayu Masyita, Ariza Yandwiputra Besari, Fathir Azzaki Iradata, Lucy Arianie

**Affiliations:** a Research Center for Vaccine and Drugs, National Research and Innovation Agency (BRIN), Raya Bogor St No 32, Cibinong, Indonesia; b Center of Excellence Life Sciences, Nano Center Indonesia, Tangerang Selatan, Indonesia; c Research Center for Pharmaceutical Ingredients and Traditional Medicine, National Research and Innovation Agency (BRIN), Raya Bogor St No 32, Cibinong, Indonesia; d Research Center for Computing, Research Organization for Electronics and Informatics, National Research and Innovation Agency (BRIN), Raya Bogor St No 32, Cibinong, Indonesia; e Research Center for Nanotechnology System, National Research and Innovation Agency (BRIN), Tangerang Selatan, Indonesia; f Chemistry Department, Faculty of Mathematics and Natural Sciences, Jakarta State University (UNJ), Jakarta, Indonesia

**Keywords:** Emodin, Hepatoprotective, HepG2, Molecular docking, Molecular dynamics, Network pharmacology

## Abstract

**Background:**

Emodin exhibits various pharmacological activities, including hepatoprotective effects. However, its clinical application is limited by poor absorption and low oral bioavailability. This study aimed to optimize emodin through benzoylation and to assess the hepatoprotective potential of the resulting derivatives integrating bioinformatics and *in vitro* methods.

**Methods:**

We conducted network pharmacology, molecular docking and molecular dynamics (MD) simulations to predict potential targets and interactions of benzoylated emodin derivatives. The derivatives were synthesized and evaluated for cytotoxicity using the MTT assay. The hepatoprotective effects were assessed *in vitro* using a paracetamol-induced HepG2 cell injury model.

**Results:**

Network pharmacology analysis and gene expression profiling identified four major targets―*HSP90AA1*, *MAPK14*, *RELA*, and *PRKACA*―as key liver disease-related targets of emodin and its derivatives. Molecular docking study revealed that the benzoylated emodin derivatives exhibited generally lower (more negative) binding energies compared to emodin across all four targets. MD simulations confirmed that the complex *RELA* with these derivatives is more stable than with emodin. These findings were consistent with *in vitro* assays, emodin and its derivatives increased cell viability or protected the cells up to 60–70%.

**Conclusion:**

This study provides a comprehensive evaluation of benzoylated emodin derivatives as potential hepatoprotective agents. The findings suggest that these derivatives exhibit binding affinity toward important targets related to cirrhosis, reduced toxicity to cells, and enhanced the efficacy in protecting the liver *in vitro* liver injury model.

## 1. Introduction

The liver is a vital organ responsible for physiological processes, including bile secretion, synthesis of essential proteins, nutrient storage, and metabolism [1]. Additionally, the liver serves as the principal site for the drug metabolism, where cytochrome P-450 enzymes convert pharmaceuticals into active or inactive metabolites [2]. Enzymes like reduced glutathione (GSH) facilitate these reactions, eliminating harmful substances and detoxifying endogenously produced free radicals. Reduced Glutathione S-transferases (GST) activity leads to upregulation of oxidative defense systems, crucial during inflammation [3,4].

Liver diseases represent a major global health burden and are among the leading causes of mortality worldwide [5]. Hepatotoxicity involves complex molecular pathways that remain poorly understood. However, it supports the hypothesis that oxidative stress causes an inflammatory cascade and organ damage by programmed cell death [6]. Many liver diseases can progress to cirrhosis, which is defined by severe liver dysfunction and complications if not treated promptly and effectively [7]. It emphasizes the critical need for novel therapeutic strategies to manage and prevent liver damage. The development of natural hepatoprotective agents represents a promising strategy for mitigating liver diseases, owing to their potential efficacy and favorable safety profiles [8].

Emodin (6-methyl-1,3,8-trihydroxyanthraquinone) is a natural anthraquinone derivative with medicinal potential. It has anti-inflammatory, antibacterial, oxidative stress inhibition, hepatoprotective, and anti-fibrotic effects [9]. *In vivo* studies have demonstrated that emodin can be safely administered to both male and female mice at doses of 20, 40, and 80 mg/kg over a 12-week period [10]. Furthermore, emodin administration in CCl_4_-induced liver injury mouse models resulted in significant reductions in liver function enzymes and showed promising effects on liver morphology [11].

Despite its promising pharmacological properties, the therapeutic application of emodin is significantly constrained by its poor intestinal absorption, rapid systemic clearance, and low oral bioavailability [12]. Following oral dosing, emodin was undetectable in serum. It is suggested that first-pass metabolism may account for the decreased bioavailability of emodin (<3%) [13]. Molecular modification strategies, such as functional group derivatization, are commonly employed to improve solubility, metabolic stability, and bioavailability while potentially reducing toxicity [14]. This investigation was previously conducted with meptazinol, a centrally acting opioid analgesic, which had been modified into a benzoyl ester form and demonstrated enhanced absorption efficacy in the rat gastrointestinal tract [15].

The present study aimed to synthesize and evaluate benzoylated emodin derivatives. The benzoyl ester forms of emodin were predicted to enhance hydrophobicity and more readily traverse the hydrophobic barrier of the cell membrane, allowing them to reach and interact with target receptors. We hypothesized that these derivatives would demonstrate superior hepatoprotective activity compared to native emodin. We employed an integrated approach: bioinformatics analysis to identify liver cirrhosis-related genetic targets, followed by functional enrichment and protein-protein interaction (PPI) analysis to understand gene interconnectivity. Molecular docking and dynamics simulations were conducted to assess the binding affinities of compounds to relevant target proteins, and the findings were validated through *in vitro* assays using a paracetamol-induced HepG2 injury model.

## 2. Materials and methods

### 2.1. Bioinformatics analysis/network pharmacology

#### 2.1.1. Target identification and integration

**Fig. 1:**
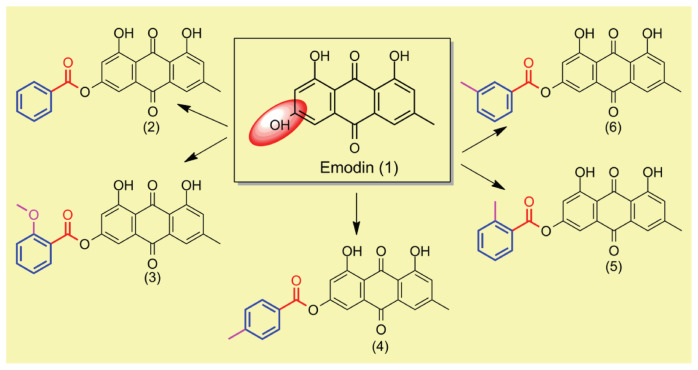
Modification of emodin.

The design of benzoylated emodin derivatives is illustrated in Fig. 1. Liver cirrhosis–associated genes were retrieved from GeneCards, Mala-Cards, and DisGeNET using the keyword “Liver Cirrhosis” [16–18]. Simultaneously, compound-related targets were predicted using TargetNet, PharmMapper, and ChemMapper, applying thresholds of >0.6 for TargetNet and PharmMapper, and a 3D similarity >0.85 for ChemMapper [19–21]. All gene sets were merged and filtered for redundancy. The intersection of compound and disease targets was determined using Venn analysis to identify shared targets for downstream network construction.

#### 2.1.2. Network construction and hub gene identification

Overlapping targets were submitted to STRING (https://string-db.org/) to construct a PPI analysis with *Homo sapiens* selected and a minimum interaction score >0.9 [22]. The resulting network was visualized using Cytoscape v3.9.1 [23], and topological analysis was performed to identify hub genes based on node centrality.

#### 2.1.3. Functional and expression analysis

Gene Ontology (GO) and KEGG pathway enrichment analyses were conducted via DAVID (https://david.ncifcrf.gov/tools.jsp) with FDR-corrected *p* < 0.05 to identify enriched biological processes and pathways [24,25]. Results were visualized using the bioinformatics visualization platform (http://www.bioinformatics.com.cn) [26]. To validate the clinical relevance of hub genes, their expression profiles were analyzed using UALCAN (http://ualcan.path.uab.edu/) with a significance threshold of *p* < 0.05 [27].

### 2.2. Molecular docking simulation

#### 2.2.1. Preparation of ligands and target proteins

Emodin and its benzoylated derivatives were designed to act as ligands. 3D conformations in “sdf” format were drawn and then optimized using Open Babel 3.1.1. Ligand preparation was conducted using AutoDock Tools 1.5.7 by adding polar hydrogens, merging non-polar hydrogens, assigning Gasteiger charges, detecting rotatable bonds, and managing aromaticity.

The 3D structures of target proteins were retrieved in PDB format from the RCSB Protein Data Bank (https://www.rcsb.org/). Protein preparation was also performed using AutoDock Tools 1.5.7, which included the removal of water molecules, native ligands, ions, and non-relevant chains. Subsequently, polar hydrogens were added, non-polar hydrogens were merged, and Kollman charges were assigned [28].

#### 2.2.2. Docking validation and simulation

Docking validation was performed by re-docking the native ligand into the binding site of its corresponding target protein to define the appropriate docking grid encompassing the active site [29]. The validation parameter for the docking method is the Root Mean Square Deviation (RMSD) value, which indicates the difference in structure position between the docked ligand and the native ligand bound to the protein during re-docking [30]. The docking process is considered valid if it has an RMSD value of <2 Å [31]. This threshold indicates that the docking software has accurately predicted the ligand’s placement and orientation within the binding site [32]. The docking validation is presented in Table 1. The docking process utilizes the docking validation grid, default docking parameters and Lamarckian Genetic Algorithm [33]. Protein-ligands interactions having the lowest binding affinity are visualized using Biovia Discovery Studio 2021 for 2D and 3D visualization.

### 2.3. Molecular dynamics (MD) simulations

The 3D conformation of the ligand obtained from the docking simulation was extracted from the protein–ligand complex. Preparation of ligands, proteins, and complexes followed a modified protocol from Ramadhan et al. (2023) [33]. MD simulations were performed using GROMACS for 100 ns on the MAHAMERU high-performance computing (HPC) system, operated by the National Research and Innovation Agency of Indonesia (BRIN).

### 2.4. Analysis of Lipinski’s rule of five, pharmacokinetic and toxicity

The physicochemical and pharmacokinetic properties of the compounds were analyzed using their canonical SMILES representations. These were input into the pkCSM online tool (http://biosig.unimelb.edu.au/pkcsm/prediction) [34,35] and ADMETlab 3.0 (https://admetmesh.scbdd.com) for ADMET profiling.

### 2.5. Synthesis of benzoylated emodin derivatives

Compounds 3-(2-methoxy) benzoyl emodin **(3)** and 3-*o*-toluoyl emodin **(5)** were obtained from our previous work and used without further modification. The complete synthesis and characterization details procedures are described in our original publication [36].

#### 2.5.1. Synthesis of 3-benzoyl emodin (2)

A semisynthesis of 3-benzoyl emodin was performed by diluting 3.7 mmol emodin (98%) **(1)** in THF and followed by 37 mmol dry pyridine. Benzoyl chloride (>99%) (8.6 mmol) was gently introduced through a separatory tunnel under constant stirring using a magnetic stirrer. The reaction was allowed for 1–2 h, followed by heating for 1 min. After completion, the mixture was poured into around 15 mL of water. The resulting solid was filtered, washed with HCl solution, and recrystallized using acetone to obtain a purified product as a solid yellow, yielding 59.5%. LC-MS *m*/*z*: 375.09 [M+H]^+^. FTIR (KBr, cm^−1^): 1742, 1616, 1474, 1381, 704, 758. ^1^H-NMR (500 MHz, CDCl_3_): δ 7.12 (1H, d, J = 0 Hz), 7.66 (1H, d, J = 0 Hz), 7.69 (1H, d, J = 1.95 Hz), 7.21 (1H, d, J = 1.95 Hz), 8.20 (2H, d, J = 7.75 Hz), 7.54 (2H, t, J = 7.15 Hz), 7.68 (1H, m), 2.47 (3H, s), 11.98 (1H, s), 12.23 (1H, s). ^13^C-NMR (125 MHz, CDCl_3_): δ 164.29, 117.08, 157.69, 113.78, 124.85, 149.56, 121.84, 163.02, 191.79, 114.16, 114.29, 181.44, 133.24, 135.32, 164.29, 130.58, 128.75, 128.80, 134.42, 22.44.

#### 2.5.2. Synthesis of 3-p-toluoyl emodin (4)

The synthesis was prepared by following the procedure described for 3-benzoyl emodin **(2)**. The resulting solid was filtered, washed with Na_2_CO_3_ and ethanol solutions, then recrystallized in acetone to get a pure product as a solid yellow, yielding 69.66%. LC-MS *m*/*z*: 389.10 [M+H]^+^. FTIR (CHCl_3_, cm^−1^): 1746, 1614, 1477, 1387, 849. ^1^H-NMR (500 MHz, CDCl_3_): δ 7.11 (1H, s), 7.66 (1H, d, J = 1.3 Hz), 7.68 (1H, d, J = 1.95 Hz), 7.20 (1H, d, J = 2.6 Hz), 2.47 (6H, s), 11.98 (1H, s), 12.23 (1H, s). ^13^C-NMR (125 MHz, CDCl_3_): δ 164.26, 117.08, 155.95, 111.34, 124.80, 149.49, 123.98, 163.04, 191.75, 114.13, 114.16, 181.43, 133.23, 135.28, 168.33, 145.44, 22.18, 22.44.

#### 2.5.3. Synthesis of 3-m-toluoyl emodin (6)

It was prepared by following the procedure described for 3-benzoyl emodin **(2)**. The resulting solid was filtered, washed with Na_2_CO_3_ and ethanol solutions, then recrystallized in acetone to get a pure product as a solid yellow, yielding 67.57%. LC-MS *m*/*z*: 389.10 [M+H]^+^. FTIR (CHCl_3_, cm^− 1^): 1734, 1613, 1472, 739, 689. ^1^H-NMR (500 MHz, CDCl_3_): δ 7.11 (1H, s), 7.66–7.68 (2H, d, J = 0 Hz), 7.20 (1H, d, J = 0 Hz), 7.99–8.01 (2H, m), 7.49 (1H, d, J = 7.75 Hz), 7.41–7.43 (1H, t, J = 7.1 Hz), 2.46 (6H, s), 11.99 (1H,

### 2.6. In vitro evaluation

#### 2.6.1. Cytotoxicity assay

HepG2 cells were seeded at a density of 1.5 × 10^4^ cells per well in a 96-well plate and incubated at 37 °C in a humidified atmosphere containing 5% CO_2_ for 24 h. The cytotoxicity of emodin and its benzoylated derivatives was evaluated using the MTT colorimetric assay [37] to determine the non-cytotoxic concentrations for subsequent experiments.

#### 2.6.2. Viability assessment of paracetamol (PCT)-induced HepG2

Viability assessment of PCT-induced HepG2 cells was conducted by quantifying the percentage of cell viability following the treatment with the test compounds and PCT. HepG2 cells were seeded at a density of 5 × 10^4^ cells per well in a 96-well plate and incubated at 37 °C in a humidified atmosphere containing 5% CO_2_ for 24 h. Cells were incubated for 24 h in FBS-free medium. Various concentrations of emodin and its derivatives were added and incubated for 4 h, followed by treatment with 30 mM PCT and further incubation for 24 h under the same conditions. After treatment, the medium was removed, and 0.5 mg/mL MTT reagent was added to each well and incubated for 4 h. The resulting formazan crystals were dissolved in 10% SDS and incubated overnight at room temperature. Absorbance was measured at 570 nm using an ELISA plate reader [38,39].

#### 2.6.3. Statistical analysis

Data are presented as mean ± standard deviation (SD) from three independent experiments. Statistical significance was determined using an unpaired Student’s *t*-test, with *p* < 0.05 considered statistically significant (IBM SPSS Statistics 26).

## 3. Results

### 3.1. Identifying potential targets of emodin derivatives in liver cirrhosis

**Fig. 2:**
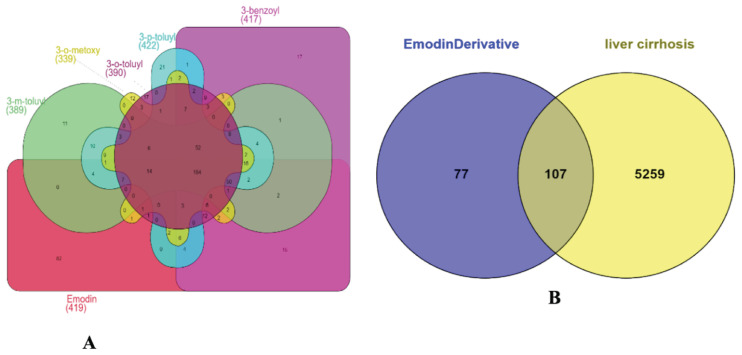
Target Tissue of Six Emodin derivative compounds (A), Target Tissue of Emodin derivative and Liver Cirrhosis.

Each of the six emodin derivatives was individually analysed to predict potential targets. A Venn diagram tool was employed to integrate the results, identifying 184 main targets representing all emodin and its derivatives (Fig. 2A). By combining gene lists from the database and removing duplicates, a total of 5366 proteins associated with liver cirrhosis were identified. The intersection of these and the 184 predicted targets of emodin derivatives resulted in 107 overlapping proteins (Fig. 2B), suggesting potential targets of emodin relevant to liver cirrhosis.

**Fig. 3:**
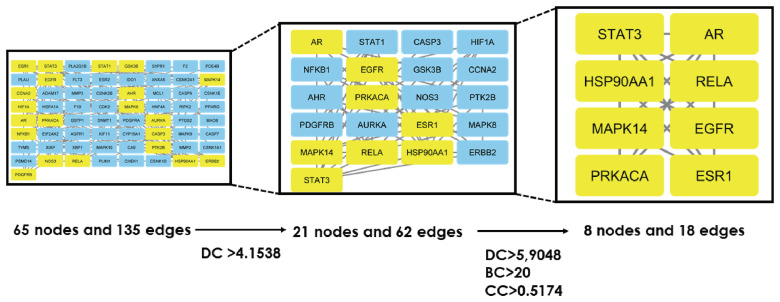
The protein-protein interaction associated with liver cirrhosis from topology Filtering Process.

The PPI analysis of 107 targets was conducted using STRING analysis. After removing unconnected genes, 65 nodes and 135 edges were obtained. The Cytoscape plugin CytoNCA was utilized for network centrality analysis. In the first stage, genes are selected with a Degree Centrality (DC) value greater than 4.1538 (average degree data value). In the second stage, genes were selected with a Degree Centrality (DC) value > 5.9048 (average degree data value), Betweenness Centrality (BC) > 20 (average BC data value), and Closeness Centrality (CC) > 0.5174 (average CC data value). Through this PPI enrichment analysis, eight core targets associated with liver cirrhosis were identified for further investigation (Fig. 3).

### 3.2. GO and KEGG enrichment analysis

**Fig. 4:**
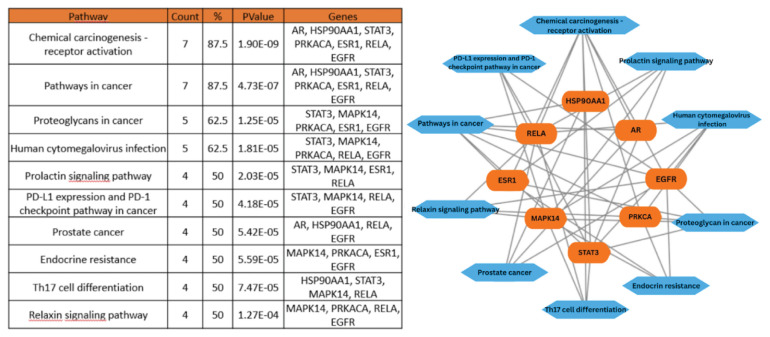
Top 10 KEGG Pathways of potential targets of Emodin derivative on Liver Cirrhosis.

KEGG pathway analysis suggests that the mechanism of emodin and its derivatives to prevent and treat cirrhosis may involve several significant signaling pathways (Fig. 4). Notably, the enrichment of the chemical carcinogenesis–receptor activation pathway implies that these compounds may modulate receptor-mediated detoxification and oxidative metabolism processes, which are commonly dysregulated during chronic liver injury. Additionally, the involvement of proteoglycans in cancer and general cancer-related pathways suggests a potential role in interfering with extracellular matrix remodeling and fibrogenesis― hallmark features of cirrhosis. These pathways often overlap with those involved in cancer due to shared mechanisms such as epithelial–mesenchymal transition (EMT), chronic inflammation, and aberrant cell proliferation.

GO analysis further indicated that the primary biological processes affected include positive regulation of transcription from RNA polymerase II promoter, signal transduction, and positive regulation of gene expression. These processes reflect the central role of emodin derivatives in modulating transcriptional responses to stress, inflammation, and injury. In particular, genes involved in the positive regulation of gene expression may contribute to hepatocyte survival and regeneration.

**Fig. 5:**
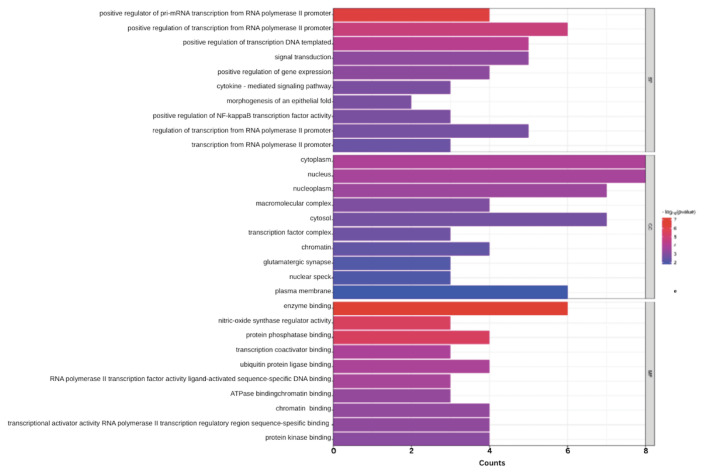
Top 10 Gene Ontology of Potential Targets of Emodin Derivatives on Liver Cirrhosis, including Biological Processes, Cellular Components, and Molecular Functions. Color represents difference -Log10 (P-value).

Cellular component analysis showed that the majority of target proteins were localized in the cytoplasm, nucleus, and transcription factor complexes, highlighting their roles in intracellular signaling and transcriptional control. Molecular function analysis revealed significant enrichment in ATP binding, protein kinase activity, and transcription factor binding, all of which are critical for mediating liver cell responses to injury and stress. These findings support the hypothesis that benzoylated emodin derivatives may exert their hepatoprotective effects by modulating intracellular signaling and transcriptional regulation in hepatocytes (Fig. 5).

Network pharmacology analysis and gene expression profiling identified four major targets― *HSP90AA1*, *MAPK14*, *RELA*, and *PRKACA*―as key liver disease-related targets of emodin and its derivatives. According to cancer OMICS data from the UALCAN, the mRNA expression of these targets was significantly upregulated in liver hepatocellular carcinoma (LIHC) patients compared to normal tissues (Table 2). This database offers valuable insights into the protein expression levels of the hub genes in cancerous specimens versus normal specimens [40].

### 3.3. Molecular docking and interactions analysis

**Fig. 6:**
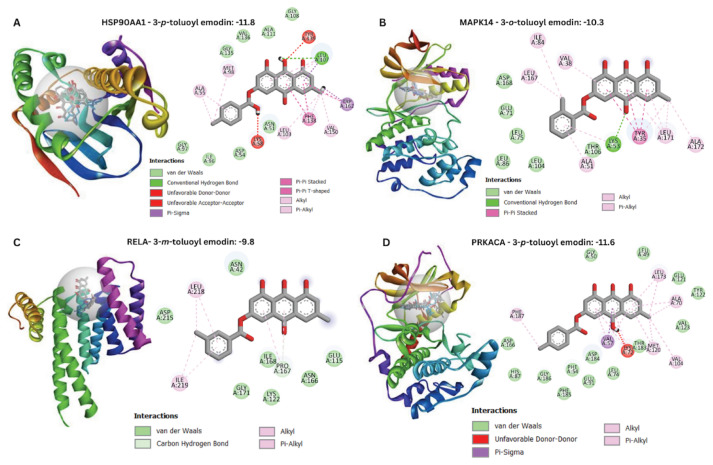
Docking analysis of the top-selected compounds against the protein targets. (A) Binding site and 2D interaction of compound (**4**) with HSP90AA1. (B) Binding site and 2D interaction of compound (**5**) with MAPK14. (C) Binding site and 2D interaction of compound **(6)** with RELA. (D) Binding site and 2D interaction of compound (**4**) with PRKACA. All emodin derivatives bind at the same site as the native ligand (blue) within the binding pocket (highlighted by a light grey circle).

A molecular docking simulation was performed to further evaluate the binding affinities of the four primary targets with six emodin and derivative structures. As shown in Fig. 6, all ligands were successfully docked at the same binding sites as the native ligands for each protein. The binding energy and molecular interactions are summarized Table 3. The result indicated that emodin and its derivatives exhibit better binding energy values than the native ligands for *HSP90AA1, MAPK14*, and *RELA*, except for *PRKACA*.

TYR139 and TRP162 were identified as crucial residues in the binding site of emodin derivatives with *HSP90AA1*. TYR139 plays a key role in stabilizing ligand-protein interactions through conventional hydrogen bonding, consistent with previous studies highlighting its significance in the binding affinity of emodin derivatives [41–43]. Similarly, TRP162, which also serves as the binding site of the native ligand, was found to interact with the emodin-based ligands via Pi-sigma interactions. This interaction was consistently observed across nearly all emodin derivatives. The essential role of TRP162 in HSP90AA1 function has also been confirmed in earlier studies [44].

Within the binding pocket of *MAPK14* with native ligands, LYS53 was observed as a crucial amino acid for binding with proteins [45–47]. Our results also confirm this finding, as LYS53 plays a key role in the docking of emodin derivatives through hydrogen bonding and Pi-cation interactions. However, only the emodin ligand did not bind to LYS53 and exhibited a lower binding affinity (− 8.1 kcal/mol). This suggests that modifying the structure of emodin enhances its binding interactions, potentially improving its stability and affinity for *HSP90AA1*.

A previous study reported that LEU218 and ILE219 stabilize ligand binding with *RELA* [48]. In our results, both residues were observed interacting with emodin derivatives, forming Pi-sigma and Pialkyl interactions, except in emodin and **(4)**. This further supports that modifying emodin enhances its ability to bind with target proteins.

### 3.4. Molecular dynamics analysis

**Fig. 7:**
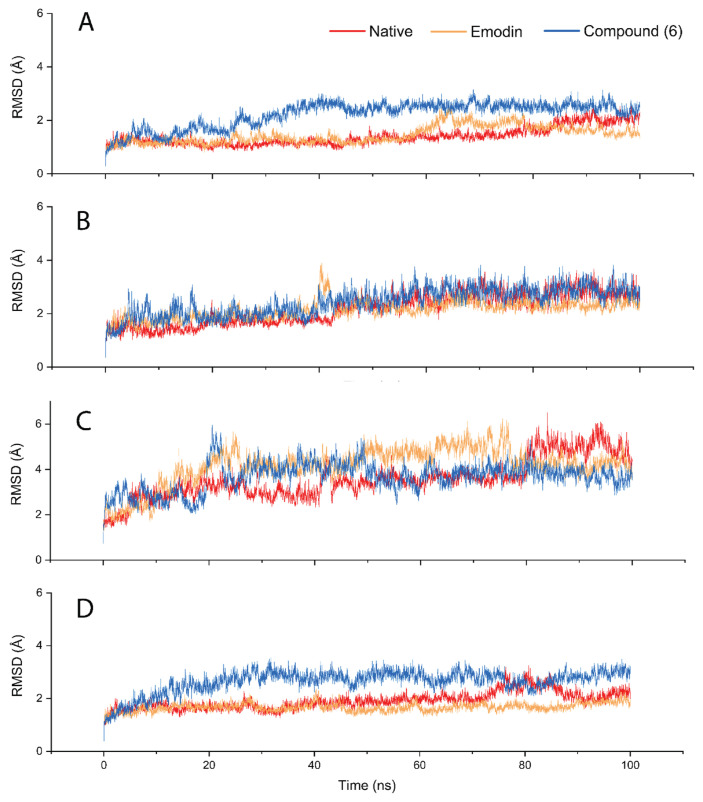
RMSD Profiles of MD simulations of HSP90AA1 (A), MAPK14 (B), RELA (C), and PRKACA (D).

**Fig. 8:**
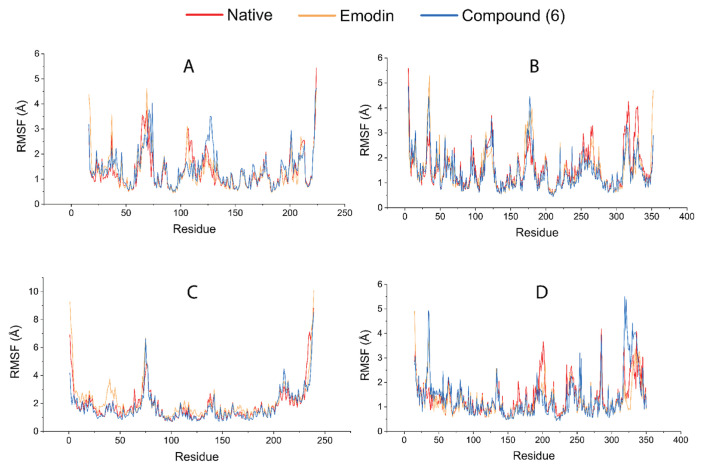
RMSF Profiles of MD simulations of HSP90AA1 (A), MAPK14 (B), RELA (C), and PRKACA (D).

The interactions between the target with its native ligand, emodin, and compound (**6**), obtained from molecular docking, were further validated through MD simulations to assess interaction stability over time. Figs. 7 and 8 show the analysis of the RMSD and RMSF profiles based on 100 ns of MD simulations. The RMSD values for *HSP90AA1*, *MAPK14*, and *PRKACA* complexes ranged from ~1 to 3 Å and stabilized after 20 ns. In *HSP90AA1* and *PRKACA* complexes, compound **(6)** showed greater deviation than emodin and the natural ligand, while in *MAPK14* complexes, all three ligands displayed similar, stable fluctuations around 2–3 Å. In *RELA* complexes, The RMSD values ranged from ~1 to 6 Å with compound (**6**) exhibited better stability than emodin and natural ligand after 80 ns. However, RMSF analysis showed minor spikes at residue TYR139 in *HSP90AA1*-compound (**6**) complex and stable fluctuations at residue ASN42 in *RELA-*compound (**6**) complex These findings are consistent with the molecular docking results, where hydrogen bonds are formed. Overall, no major differences in RMSF profiles were observed, suggesting that all protein–ligand complexes maintained structural stability.

### 3.5. Physicochemical and ADMET properties prediction

An evaluation of physicochemical parameters was conducted on emodin and its benzoylated derivatives to assess their compliance with Lipinski’s rules, with the results summarized in Table 4. The data indicate that the designed compounds meet the criteria for the development of small-molecule therapeutic candidates. Furthermore, we analyzed key parameters including human intestinal absorption (HIA), Caco-2 cell permeability, volume of distribution at steady state (VDss), blood-brain barrier (BBB) permeability, CYP1A2 substrate classification, and total clearance to characterize the compound’s ADME profile (Table 5). According to the pkCSM predictive model, compounds exhibiting an intestinal absorption rate below 30% are classified as poorly absorbed. Caco-2 permeability is considered high when the predicted value exceeds 0.90. The VDss is categorized as low when below 0.71 L/kg (log VDss < −0.15) and high when above 2.81 L/kg (log VDss > 0.45). A log BB value greater than 0.3 suggests that a compound is capable of effectively crossing the blood-brain barrier (BBB), whereas values < −1 indicate limited brain penetration. Emodin has an HIA value of 69.27%, whereas its derivatives exhibit significantly higher values, ranging from 80% to 90%. This indicates that the derivatives are more efficiently absorbed through the intestinal lining, potentially resulting in improved bioavailability compared to the parent drug. Additionally, emodin derivatives show high Caco-2 permeability values, further supporting their enhanced oral absorption. The VDss values for the derivatives are either negative or lower than those of emodin, suggesting a greater concentration in plasma rather than in tissues. All emodin derivatives, except compounds 2 and 3, are substrates of CYP1A2, indicating that they can be metabolized by cytochrome P450 enzymes. Lower total clearance values imply that the derivatives have higher bioavailability and slower metabolic elimination. Moreover, the derivatives demonstrate reduced toxicity compared to emodin.

### 3.6. Benzoylated emodin derivatives

The structures of the synthesized products were confirmed using MS, FTIR, ^1^H NMR, and ^13^C NMR spectroscopy. The reactions of emodin **(1)** with benzoyl chloride derivatives and pyridine were monitored and successfully yielded benzoylated emodin derivatives (**2**)–(**6**). The FTIR spectra showed a characteristic band for the ester carbonyl group at around 1740 cm^−1^, indicating the presence of a C=O stretching vibration typical for ester functionalities. The presence of the aromatic ring was confirmed by two characteristic absorption bands at 1616 and 1473 cm^−1^, corresponding to C=C stretching vibrations in the aromatic system. While the presence of other groups substituted on the ring was consistent with their respective structures. In the ^1^H NMR spectra, the disappearance of a single peak in the 5–12 ppm range—attributed to hydroxyl protons—along with the appearance of a new carbonyl carbon signal at around 164 ppm in the ^13^C NMR spectrum, confirmed the successful esterification of emodin.

### 3.7. In vitro evaluation

**Fig. 9:**
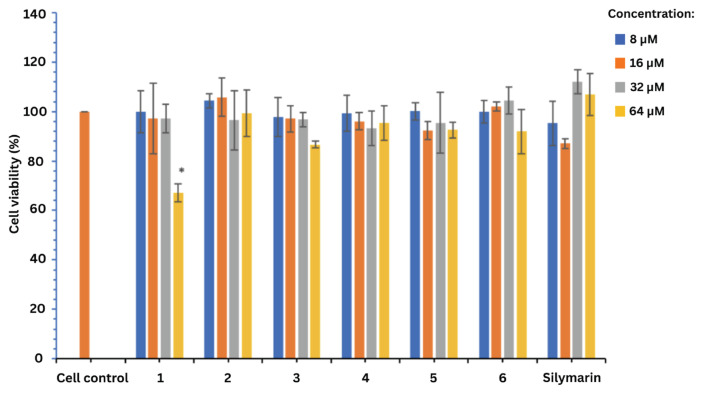
Cytotoxicity effect of Emodin and its derivatives in HepG2 cells. HepG2 cells were incubated for 24 h in the absence or presence of a sample at concentrations ranging from 0 to 64 μM, and the percentage of cell viability was evaluated using an MTT method. The data were reported as the means and standard deviations of independent experiments. *P < 0.05 vs untreated cell.

The percentage of HepG2 cell viability following treatment with emodin, its benzoylated derivatives, and silymarin at four different concentrations is shown in Fig. 9. All compounds demonstrated cell viability above 80% in HepG2 cells across all tested concentrations compared to the cell control, with the exception of emodin at 92 μM. Cells were treated with varying doses of compounds for 24 h, and cell viability was assessed using the MTT assay. The experiments were conducted in triplicate. *P < 0.05 compared to cell control.

**Fig. 10:**
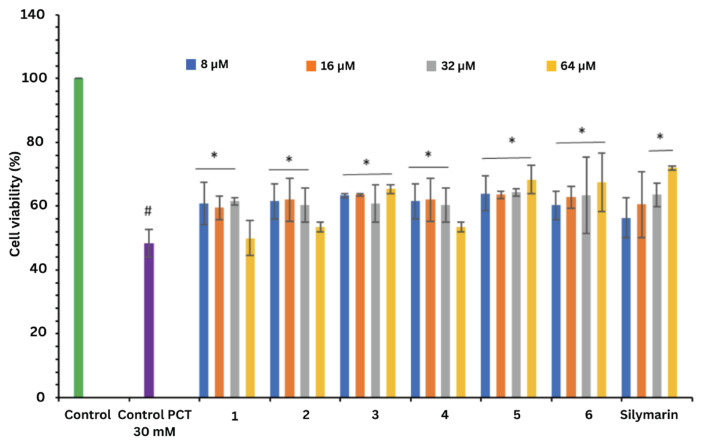
The hepatoprotective effect of Emodin and its derivatives in paracetamol induced-HepG2 cells. HepG2 cells were treated with samples for 4 h of incubation in various concentrations (0–64 μM) in free FBS-DMEM, followed by the addition of 30 mM paracetamol (PCT). After 24 of incubation, the percentage of cell viability was conducted using an MTT method. The data were presented as the means ± standard deviations of three independent experiments. *P < 0.05 vs control; *P < 0.05 vs control PCT 30 mM.

The percentage of HepG2 cell viability following treatment with emodin, benzoylated emodin derivatives, and silymarin across four concentration ranges is shown in Fig. 10. All tested samples significantly promote cell proliferation compared to the control treated with 30 mM PCT.

## 4. Discussion

In the present study, we structurally modified emodin by replacing its hydroxyl group with a benzoyl derivative to enhance its hepatoprotective efficacy and bioavailability. The ADMET prediction data indicate that the benzoylated emodin derivatives exhibit superior characteristics. They are generally regarded as meeting the criteria of Lipinski’s Rule, a framework utilized to evaluate drug-likeness. The benzoylated emodin derivatives show advantageous absorption in the mucosa and intestines, display wider distribution in plasma, are metabolized by CYP1A2, have increased bioavailability, and are less toxic.

These findings are consistent with a recent study by Zhou et al. (2023), who conducted an in-depth investigation of emodin metabolism both *in vitro* and *in vivo*. They reported that emodin exhibits poor bioavailability, with an absolute bioavailability of 3.197 ± 0.714%. Approximately 56% of the compound remained unabsorbed and was primarily excreted unchanged in the feces. The absorbed fraction was rapidly metabolized into hydroxylated and glucuronidated forms. The study identified liver enzymes CYP1A2, CYP2E1, UGT1A1, UGT1A9, and UGT2B7 as the primary contributors to emodin metabolism in rat liver microsomes, resulting in the formation of hydroxylated (ω-hydroxyemodin and 2-hydroxyemodin) and glucuronidated (emodin-3-O-glucuronide) metabolites. Glucuronidated metabolites were detected in rat plasma following oral administration but were absent after intravenous dosing. Thus, the low oral bioavailability of emodin may be due to its rapid metabolism to glucuronidated metabolites via hepatic phase II metabolism after oral administration. Furthermore, the authors proposed that the extensive metabolism of emodin after oral administration may contribute to the formation of reactive, potentially toxic metabolites [49].

A network pharmacology analysis was conducted to identify appropriate and linked target proteins between emodin derivatives and liver cirrhosis. *HSP90AA1, MAPK14, RELA, and PRKACA* are involved in critical pathways associated with inflammation, apoptosis, and cellular stress responses, making them promising therapeutic targets for cirrhosis treatment [50]. Among these, *HSP90AA1* is a molecular chaperone known to stabilize multiple client proteins involved in hepatic stress, inflammation, and fibrosis. It is upregulated in cirrhotic liver tissues [51], and pharmacological inhibition of *HSP90AA1* has been proposed to disrupt fibrogenic signaling pathways and mitigate liver injury. Emodin derivatives with high affinity for *HSP90AA1* may thus suppress disease-promoting pathways at a proteostatic level. *MAPK14* (p38 MAPK) is a critical regulator of hepatic inflammation and fibrogenesis. It plays a role in transducing extracellular stress signals and promoting pro-inflammatory cytokine expression. Inhibition of *MAPK14* has been shown to attenuate liver damage in animal models of acute liver failure and alcohol-induced injury, making it a promising target for antifibrotic and hepatoprotective therapies [52,53]. Furthermore, *RELA*, a subunit of the NF-κB complex, governs the transcription of pro-inflammatory and survival-related genes. Sustained NF-κB activation is a hallmark of chronic liver diseases, contributing to hepatic inflammation and progression to fibrosis. *RELA* inhibition has been reported to reduce liver inflammation in models of metabolic liver disorders, including high-carbohydrate-diet-induced fatty liver disease [54]. Although *PRKACA*, the catalytic subunit of protein kinase A, has been implicated in Fibrolamellar Hepatocellular Carcinoma through gene fusions [55], our results showed weak binding by emodin derivatives to this protein, suggesting limited therapeutic potential via this pathway without further structural optimization.

Our *in silico* study showed that the binding energy of benzoylated emodin derivatives is generally more negative than the binding energy of emodin against the four target proteins. This suggests that, regarding the activity, benzoylated emodin compounds at position C3 exhibit superior efficacy compared to unmodified emodin. It is indicated that the hydroxyl group at C3 is not a pharmacophore, allowing for modification to enhance activity and improve physicochemical or ADMET qualities. Its derivatives also showed comparable dynamic stability to emodin, reinforcing their suitability as lead compounds.

Benzoylated emodin derivatives can be synthesized through the esterification of emodin. Esters can be synthesized via the reaction of alcohols with acyl chlorides. This reaction is reversible and is hence favored. Given that acyl chlorides react with water, this reaction is conducted in an anhydrous (water-free) environment. The esterification reaction of emodin can yield diverse molecules due to the presence of three hydroxyl groups. Nevertheless, the 1,8-dihydroxy groups of emodin are positioned near the 9-carbonyl group, resulting in the formation of two intramolecular hydrogen bonds, while the 3-hydroxyl group is far from the 9- and 10-carbonyl groups. The reactivity of the 3-hydroxy group surpasses that of the 1- and 8-hydroxy groups, and it can be appropriately organized under certain conditions [56]. Nonetheless, the potential for the substitution of the hydroxyl group persists. This results in the yield of the benzoylated emodin derivatives produced exclusively at C3 being around 59%. The production of toluyl emodin exceeds that of benzoyl emodin under the same conditions. The methyl group, as an electrondonating entity, facilitates the stabilization of the reaction, hence enhancing its ease.

The hepatoprotective efficacy of emodin and its derivatives was assessed using HepG2 hepatocarcinoma cells as an i*n vitro* model. These cells were selected due to their high proliferation rate and enhanced stability of drug-metabolizing enzymes relative to primary hepatocyte cultures. The samples were initially assessed by cytotoxicity assay in HepG2 cells using the MTT method. These non-toxic concentrations were used for hepatoprotective assay. Emodin is recognized for its cytotoxic capabilities, which are utilized as an anticancer agent against several cell types, including HepG2 cells. The results indicated that all tested compounds did not inhibit cell proliferation across all concentration ranges, except for emodin at 64 μM. At this concentration, emodin significantly reduced the percentage of cell viability to approximately 30% compared to the untreated control. These findings were used to determine the concentration of compounds in the hepatoprotective assay.

HepG2 cells are vital in metabolism and toxicity investigations concerning hepatotoxic pharmaceuticals, including paracetamol (PCT) [57]. PCT causes drug-induced liver injury in a predictable, dose-dependent way and is toxic to the liver in and of itself. CYP2E1 in the liver metabolizes a minor fraction of PCT into the highly reactive and hazardous electrophilic arylated metabolite, N-acetylp-benzoquinoneimine [58]. Following 24 h of PCT induction in HepG2 cells, significant cellular damage or mortality was observed, with cell viability decreasing to approximately 48% compared to cells without induction. Treating the cells with emodin and its derivative before the PCT-induction can diminish the effect of PCT to inhibit the proliferation of the cells. Emodin and its derivatives increased cell viability or protected the cells up to 60–70%. Emodin and its derivatives exhibited significant hepatoprotective effects at concentrations of 8, 16, and 32 μM. However, at 64 μM, emodin, compound **(2)**, and compound **(4)** showed a reduced protective effect, likely due to cytotoxicity at higher concentrations. In contrast, compounds **(5)** and **(6)** maintained their hepatoprotective activity even at 64 μM. These findings suggest that emodin derivatives, starting from a concentration as low as 2 μM, provide superior protection against PCT-induced cytotoxicity in HepG2 cells compared to emodin alone. Silymarin (64 μM), as a positive control, also exhibited strong protective effects, resulting in 70% cell viability. This aligns with previous studies reporting that silymarin protected HepG2 cells from PCT-induced toxicity, achieving a viability of 75.83% [59].

Overall, this study offers a comprehensive evaluation of benzoylated emodin derivatives as potential anti-cirrhotic agents. These derivatives exhibit enhanced molecular interactions with key cirrhosis-related targets, reduced cytotoxicity, and improved hepatoprotective efficacy in an *in vitro* model of liver injury. Therefore, more investigation is required to elucidate the precise mechanism of action of benzoylated emodin, such as analysis of biochemical parameters, oxidative stress indicators, and gene and protein expression, both *in vitro* and i*n vivo*. Furthermore, it is necessary to characterize its pharmacokinetic and pharmacodynamic features and determine the optimal dosage in animals and humans through pre-clinical and clinical studies.

## Figures and Tables

**Table 1:** Docking validation of protein target and native ligands, redocked (purple) vs. original (light grey).

Gene	PDB ID	Position	RMSD (Å)	Superimpose
*HSP90AA1*	4BQG	x = 2.257 y = 13.488 z = 23.501	1.407	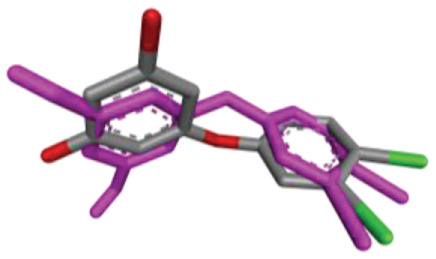
*MAPK14*	1OZ1	x = 26.178 y = −6.62 z = 12.746	0.694	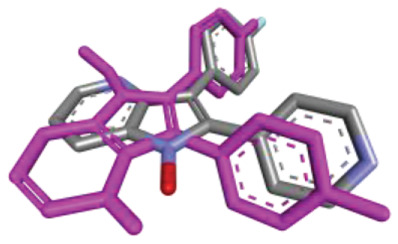
*RELA*	7BIW	x = −16.389 y = −21.23 z = 0.781	1.113	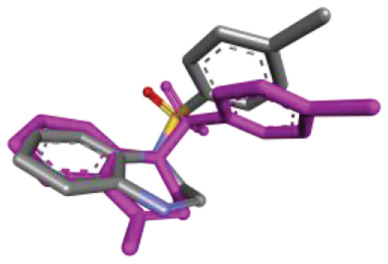
*PRKACA*	7Y1G	x = −74.128 y = −12.252 z = 12.588	0.553	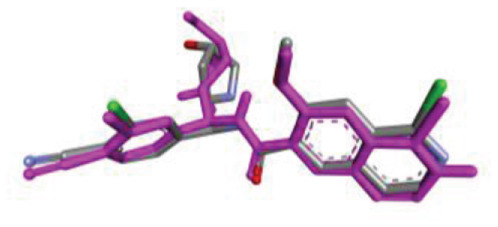

**Table 2:** Gene expression analysis of protein target in liver cirrhosis.

Protein	Expression levels	Statistical significance
*HSP90AA1*	Up Regulated	*1.624* × *10*^−^*^12^*
*STAT3*	Down Regulated	*4.246* × *10*^−^*^1^*
*MAPK14*	Up Regulated	*1.624* × *10*^−^*^12^*
*AR*	Down Regulated	*7.174* × *10*^−^*^4^*
*RELA*	Up Regulated	*1.624* × *10*^−^*^12^*
*PRKACA*	Up Regulated	*1.468* × *10*^−^*^8^*
*ESR1*	Down Regulated	*1.110* × *10*^−^*^16^*
*EGFR*	Down Regulated	*2.492* × *10*^−^*^1^*

**Table 3:** The binding energy of emodin derivative and native ligands.

Ligand	HSP90AA1		MAPK14		RELA		PRKACA	
			
	Binding Affinity	H-bond	Binding Affinity	H-bond	Binding Affinity	H-bond	Binding Affinity	H-bond
(1)	−10.7	TYR139 (2.71 Å)	−−8.1	MET109 (3.30 Å)	−8.1	LYS122 (3.19 Å)	− 10	LYS72 (2.99 Å)
(2)	−11.2	TYR139 (2.78 Å)	−9.9	LYS53 (2.84 Å)	−9.4	ASN21 (2.92 Å)	−11.1	LYS72 (3.08 Å) THR183 (3.50 Å)
(3)	−10.7	TYR139 (2.70 Å)	−9.8	LYS53 (2.93 Å)	−8.7	–	−11	LYS72 (3.01 Å)
(4)	−11.8	TYR139 (2.70 Å) LYS58 (3.18 Å)	−10.3	LYS53 (2.98 Å)	−9.8	ASN42 (2.96 Å)	−11.6	LYS72 (2.99 Å)
(5)	−11.3	TYR139 (2.78 Å)	−10.3	LYS53 (2.94 Å)	−9.4	ASN42 (2.90 Å)	−11.6	LYS72 (2.84 Å) THR51 (3.39 Å)
(6)	−11.6	TYR139 (2.75 Å)	−10.3	LYS53 (3.04 Å)	−9.8	ASN42 (3.00 Å) 1LE168 (3.36 Å)	−11.4	LYS72 (3.27 Å)
Native	−8.2	ASN51 (3.15 Å) SER52 (2.29 Å) ASP93 (2.34 Å)	−7.9	LYS53 (2.77 Å) MET109 (3.00 Å)	−7.9	ASN42 (3.11 Å)	−11.8	LYS72 (3.33 Å) ASP184 (2.86 Å) GLY55 (2.94 Å) GLU121 (2.69 Å)

**Table 4:** Predicted physicochemical properties of Emodin derivative compounds.

Compounds	Molecular Weight	LogP	Acceptors	Donors
(1)	270.24	1.88722	5	3
(2)	374.348	3.40082	6	2
(3)	404.374	3.40942	7	2
(4)	388.375	3.70924	6	2
(5)	388.375	3.70924	6	2
(6)	388.375	3.70924	6	2

**Table 5:** Predicted pharmacokinetic properties of emodin derivative compounds.

Compound	Human Intestinal Absorption (%)	Caco2 permeability (log Papp in 10^−6^ cm/s)	VDss (human) (log L/kg)	BBB permeability (log BB)	CYP1A2 substrate	Total Clearance (log mL/min/kg)	AMES toxicity	Hepatotoxicity	Oral Rat Acute Toxicity (LD_50_)
1	69.277	0.594	0.289	−0.863	+++	0.39	Yes	No	2.198
2	81.12	0.922	−0.864	−0.288	− −	0.215	No	Yes	2.061
3	90.085	0.488	−0.906	−0.382	− − −	0.131	No	Yes	2.222
4	90.901	0.595	−0.813	−0.17	+++	0.144	No	No	2.141
5	81.528	0.958	−0.764	−0.292	++	0.227	No	Yes	2.126
6	90.845	0.597	−0.821	−0.166	+	0.151	No	No	2.138
